# Hypotheses About the Relationship of Cognition With Psychopathology Should be Tested by Embedding Them Into Empirical Priors

**DOI:** 10.3389/fpsyg.2018.02504

**Published:** 2018-12-10

**Authors:** Michael Moutoussis, Alexandra K. Hopkins, Raymond J. Dolan

**Affiliations:** ^1^Max Planck Centre for Computational Psychiatry and Ageing, University College London, London, United Kingdom; ^2^Wellcome Centre for Human Neuroimaging, University College London, London, United Kingdom

**Keywords:** random effect analysis, empirical priors, computational modeling, maximum likelihood, Bayesian statistics, item response theory

Mechanistic hypotheses about psychiatric disorders are increasingly formalized as computational models. Model parameters, characterizing for example decision-making biases, are hypothesized to correlate with clinical constructs. This is promising (Moutoussis et al., 2016), but here we comment that techniques used in the literature to minimize noise in parameter estimation may not be helpful. In addition, we point out related pitfalls which may lead to questionable research practices (Sijtsma, 2015). We advocate incorporating cross-domain, e.g., psychopathology-cognition relationships into the parameter inference itself.

Maximum-likelihood techniques often provide noisy parameter estimates, in the sense of total error over an experimental group. In addition, in large studies brief tasks are often used, providing little data per participant. However, individual parameter estimation can be improved by using empirical priors (Efron, 2012). Here, parameter estimates are informed by, or conditioned upon, the population distribution that a case comes from. For an individual *j* with parameters θ*j* coming from a population distribution *p*_*pop*_:

(1) $$\begin{array}{r} p\left(\theta_{j}|data\right)\;\alpha p\left(\theta_{j},data\right)=p\left(data_{j}|\theta_{j}\right)p_{pop}\left(\theta_{j}\right) \end{array}$$

This will filter noise to shift parameter estimates nearer to the modes of *p*_*pop*_(θ). Under Gaussian assumptions,

(2) $$\begin{array}{r} p_{pop}\left(\theta \right)=N\left(\Theta_{pop},\sigma_{pop}\right) \end{array}$$

Where Θ_*pop*_ is the population mean. However, equation 2 ignores the very hypotheses that we are out to test. It is ignorant of relationships with psychiatric variables, say “anxiety” ψ_*j*_. Suppose people with “too high” θ are over-anxious, and “too low,” under-anxious. Under such a hypothesis, the above isn't just less specific, it is wrong. Estimating θ with it will suppresses the anxiety-related variability of θ, as from the point of view of the model it is indistinguishable from any other source of noise. We should allow high-anxious people to have a different mean θ than low-anxious (Gelman et al., 2013):

(3) $$\begin{array}{rcl} \mu_{\theta }\left(\psi \right) & = & m\psi +\Theta_{pop} \end{array}$$

(4) $$\begin{array}{rcl} p_{pop}\left(\theta \right) & = & N\left(m\psi +\Theta_{pop},\sigma_{pop}\right) \end{array}$$

The primary objective thus becomes estimating the credible interval of *m* relating cognition to anxiety.

Researchers may estimate parameters using:

a) Psychiatrically informed priors (a “full model”), at minimum as per equation 4.
b) No empirical priors at all, but fixed-effects, such as maximum-likelihood (ML) estimation.
c) One, psychiatrically uninformed prior. We call this a “standard fit,” equation 2.
d) Groups of participants, e.g., healthy vs. clinical populations, then using equation 2 for each group, e.g., estimating Θ_*clin*_, σ_*clin*_, Θ_*healthy*_, σ_*healthy*_.

Careful researchers often formally compare models (c) and (d), (e.g., Mkrtchian et al., 2017), but rarely use (a), which we advocate here. These methods must be used with great care, otherwise they may induce false positive results (Sijtsma, 2015). The key pitfall here is estimating parameters on the basis of a particular hypothesis and then performing “off the shelf” significance tests on the resulting parameter point estimates. For example, if a regression is included in the model, as in (a), or two distinct groups are modeled as in (d), one must be careful not to estimate statistical significance by fitting a line to the parameter point estimates in (a), or doing a between-group test in (d). As we shall see, the pitfall of using (c) alone is obtaining a false negative result, which is more of a self-defeating questionable practice.

We became aware of the importance of psychometrically dependent priors while working with empirical datasets. However, analysis of real data involves much irrelevant detail, so here we demonstrate the importance of the “full model” using synthetic data that captures essential features. As in real life, we take the estimation of θ_*j*_ from data *dj, p*(data_*j*_|θ_*j*_) in equation 1, to be noisy. We set it to be about 2–3 times as noisy over the range of psychopathology, and we explored heteroskedasticity, but this is not crucial:

(5) $$\begin{array}{r} d_{j}\sim N\left({\theta_{j}}^{true},\sigma \left(\psi_{j}\right)\right) \end{array}$$

We generated low and high psychopathology groups, both *n* = 25, by sampling ψ_*j*_ with mean +2 or −2 and sd = 1, and set Θ_*pop*_ = 4. *m* was about 0.3, so that psychopathology explained a moderate amount of parameter variability. We sampled generative parameters using equation 4, then generate data using equation 5 for 1,000 separate repetitions of the “experiment.” We fitted models (a–d) using *stan* (Carpenter et al., 2017). All models fitted the variance in equation 5 by fixed effects. We estimated *m* either through its mode and credible interval for the full model, or by linear regression in the other models. For the grouped case (d), the mean and difference in Θ_*group*_ entered a single model and the credible interval of the difference determined significance.

We found that the standard fit substantially distorted estimates of *m*, as expected from the qualitative argument above (Figure 1A, blue). Worse still, it gave ~7% more false negative results compared to the full fit (Figure 1B, green vs. blue). ML fits gave noisier parameter estimates but inferred *m* in an unbiased (Figure 1A, pink), and about in as sensitive manner, as the full fit. Grouped fits (d.) still under-estimated *m*, e.g., CI = (0.25,0.27) with generative *m* = 0.33, but did much better than the standard fit. (a–d) were also tested for false-positives but no differences of note were found (*m* = 0.0, 0.25, or 0.33). In addition, hypotheses about more than one parameters could be conveniently modeled by the multivariate version of Equation 1–4.

**Figure 1:**
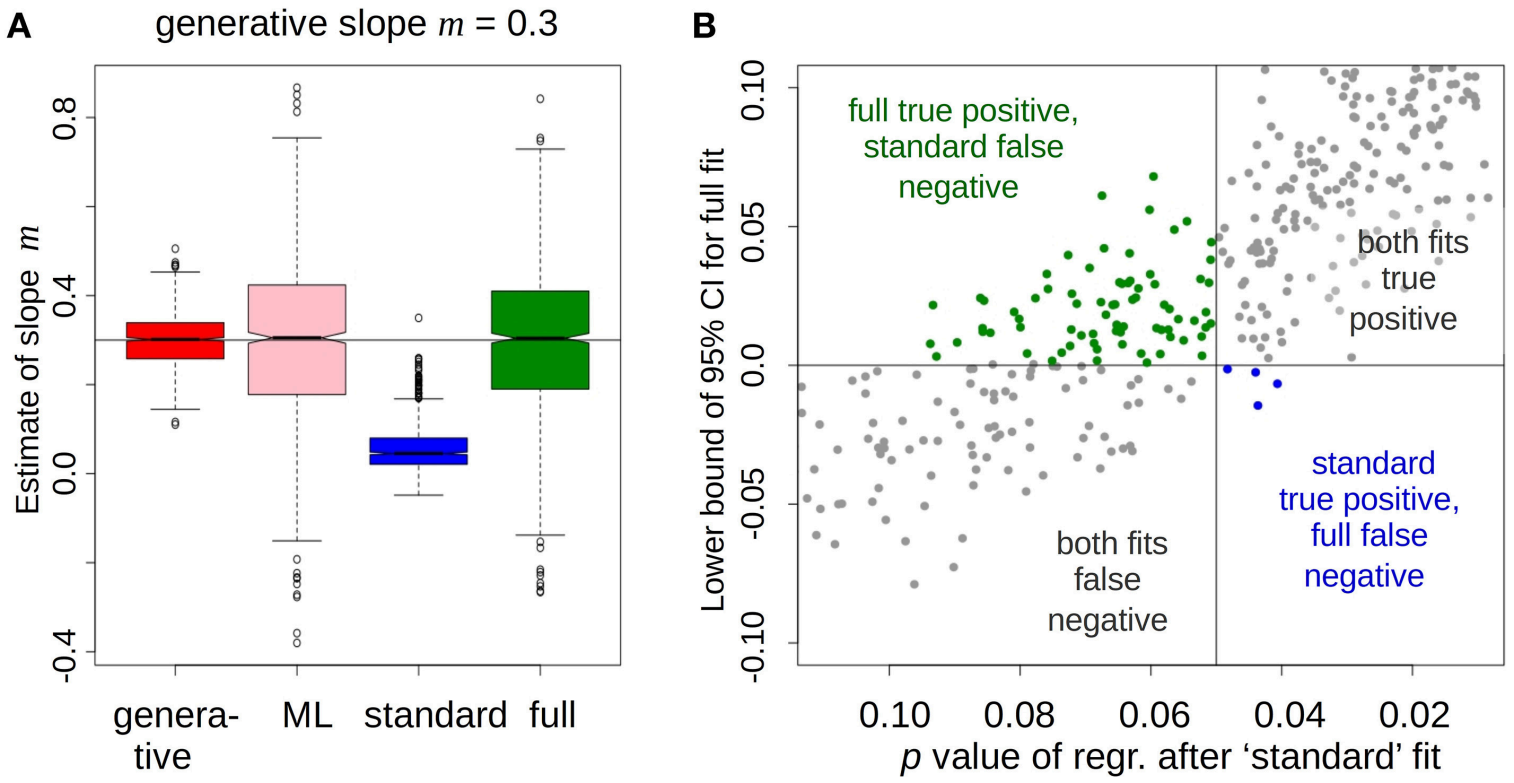
Full fit, including regression of parameter vs. psychometric variable, gives an unbiased estimate of the relationship and an increased number of true positives compared to the standard fit. **(A)** Standard fit (blue) strongly underestimates the slope of the relationship compared to the ground truth (red), maximum-likelihood (pink), and full (green) fits. **(B)** When the 95% credible interval (full fit, ordinate) and the *p*-value of the regression (standard fit, abscissa) of the slope are used as detection criteria, the full fit gives about 7% more true-positives than the standard (green vs. blue). Cross-hairs are at the conventional thresholds for 95% significance.

Therefore, if empirical priors are used to estimate cognitive and neural parameters, they must take account of the psychiatric hypotheses of the study. Standard, psychometrically uninformed priors distort the magnitude of relationships with unmodeled variables and can make them harder to detect. Here, maximum-likelihood fitting (or in real life a weakly regularized fit) better preserves umodeled relationships at the expense of noisier individual parameter estimates. There is, however, little point in inverting the wrong, psychometrically uninformed model. In addition, testing multiple hypotheses about relationships between psychiatric scores and neurocognitive parameters can be conveniently done within the full model.

Finally, psychometric measures ψ are themselves best seen as random variables inferred from measurements ϕ. We thus suggest using the likelihood of the psychometric measurements estimated by item response theory (IRT), as in Gray-Little et al. (1997), and treating ψ as another parameter, just like θ, in equation 1. The crucial prior expressing the hypothesized relation of ψ with θ then forms a multivariate density, equation 4 becoming:

(6) $$\begin{array}{r} \left(\theta_{j},\psi_{j}\right)\sim N\left(\mu_{\theta ,\psi },\Sigma_{\theta ,\psi }\right) \end{array}$$

Using IRT-derived likelihoods, modeling studies can take better account of the far from negligible uncertainty in psychiatric measurements. We acknowledge that the use of Bayesian statistics of the type advocated here (Gelman et al., 2013; Carpenter et al., 2017) is not as yet routine in psychology and neuroscience, and researchers wishing to make best use of such methods should not hesitate to seek input from the statistics community (Sijtsma, 2015).

## Author Contributions

MM proposed the concept, developed the computer code for simulations, and carried out the simulation work and wrote the first manuscript draft. AH contributed to the concept, carried out the empirical work and analyses on which the concept was based, helped develop the computer code and reviewed the manuscript. RD supervised MM and AH with respect to this work, provided critical feedback, reviewed, and edited the manuscript and provided management and funding for the project.

### Conflict of Interest Statement

The authors declare that the research was conducted in the absence of any commercial or financial relationships that could be construed as a potential conflict of interest.
